# Influence of age on postural control during dual task: a centre of pressure motion and electromyographic analysis

**DOI:** 10.1007/s40520-021-01888-x

**Published:** 2021-06-11

**Authors:** Enrico Roma, Stefano Gobbo, Valentina Bullo, Fabiola Spolaor, Zimi Sawacha, Federica Duregon, Gioia Bianchini, Eleonora Doria, Cristine Lima Alberton, Danilo Sales Bocalini, Lucia Cugusi, Andrea Di Blasio, Andrea Ermolao, Marco Bergamin

**Affiliations:** 1 Laboratoire Interuniversitaire de Biologie de la Motricité (LIBM), EA 7424, Univ Lyon, UJM Saint-Etienne, 42023 Saint-Etienne, France; 2 Sport and Exercise Medicine Division, Department of Medicine, University of Padova, Via Giustiniani, 2, 35128 Padova, Italy; 3 Department of Information Engineering, University of Padova, Via Gradenigo, 6, 35121 Padua, Italy; 4 Department of Medicine, University of Padova, Via Giustiniani, 2, 35128 Padova, Italy; 5 GymHub S.R.L., Spin-off of the University of Padova, Via O. Galante 67/a, 35129 Padova, Italy; 6 Physical Education School, Federal University of Pelotas, Rua Luís de Camões, 625, Pelotas, RS 96055630 Brazil; 7 Laboratorio de Fisiologia e Bioquimica Experimental, Centro de Educacao Fisica E Deportos, Universidade Federal do Espirito Santo (UFES), Av. Fernando Ferrari, 514, Goiabeiras, Vitoria, ES Brazil; 8 Department of Biomedical Sciences, University of Sassari, Viale San Pietro 43/B, 07100 Sassari, Italy; 9 Department of Medicine and Sciences of Aging, G. D’Annunzio University of Chieti-Pescara, Via dei Vestini, 31, 66013 Chieti, Italy

**Keywords:** Balance, Cognition, Fall, Elderly, Stabilometry, Emg

## Abstract

**Background:**

Dual task influences postural control. A cognitive task seems to reduce muscle excitation during a postural balance, especially in older adults (OA).

**Aim:**

The aim of this study is to evaluate the effect of three cognitive tasks on muscle excitation and static postural control in OA and young adults (YA) in an upright posture maintenance task.

**Methods:**

31 YA and 30 OA were evaluated while performing a modified Romberg Test in five different conditions over a force plate: open eyes, closed eyes, spatial-memory brooks’ test, counting backwards aloud test and mental arithmetic task. The surface electromyographic signals of Tibialis anterior (TA), Lateral Gastrocnemius (GL), Peroneus Longus (PL), and Erector Spinae (ES) was acquired with an 8-channel surface electromyographic system. The following variables were computed for both the electromyographic analysis and the posturographic assessment: Root mean square (RMS), centre of pressure (CoP) excursion (Path) and velocity, sway area, RMS of the CoP Path and 50%, 95% of the power frequency. Mixed ANOVA was used to detect differences with group membership as factor between and type of task as within. The analysis was performed on the differences between each condition from OE.

**Results:**

An interaction effect was found for Log (logarithmic) Sway Area. A main effect for task emerged on all posturographic variables except Log 95% frequencies and for Log PL and ES RMS. A main effect for group was never detected.

**Discussion and conclusion:**

This study indicates a facilitating effect of mental secondary task on posturographic variables. Non-silent secondary task causes increase in ES and TA muscle activation and a worsening in static postural control performance.

## Introduction

Postural control is a complex motor skill that results from the interaction between biomechanical constraints, movement and sensory strategies, orientation in space and cognitive processing [1]. Even if static postural control is an automatic process [2], it can require a certain level of attention in dual task condition [3–5]. If a secondary task is carried out concurrently with the postural one, attentional resources will be divided [6–9].

Performing two tasks at the same time is defined as dual task (DT) [10]. Dual-task cost (DT cost) occurs when the DT paradigm includes two tasks (e.g., a motor task and a cognitive one) [11] and it is measured as a deviation from a single task (ST) performance. Task prioritization depends on type of tasks, goal and instruction given to participants, defining concept of postural reserve and hazard estimation [12]. Older adults (OA) tend to perform worse than young adults (YA) under DT condition [13, 14], especially in the postural task [10]. However, part of the explication is that postural sway has been shown to increase with ageing [13], showing a worsening in static postural control. If the postural task is not threating, prioritization will favour the concurrent one [15]. Increasing difficulty of postural task enhances its DT cost but does not cause a reduction in performance of the cognitive concurrent task [16]. The present phenomena can be partially explained by bottleneck theory [17]. Moreover, degenerative processes that culminate in cognitive impairment could explicate reduction of attentional resources in older adults [10]. It has been demonstrated that OA are unable to select relevant information present in the environment, using all of them [18]. However, there is no consensus on how this could affect postural control [13, 14, 19]. Older Adults attempt to decrease postural sway by increasing ankle stiffness to counteract the inability to quickly respond to perturbations [5, 16, 19–21], by increasing co-contraction of agonist–antagonist muscles of the lower leg [5] and by a tighter neuromuscular control [16, 22]. The first mechanism could be considered as a strategy to make postural control more automatic [20, 21], even if they are energetically inefficient [23]; the second one requires attentional resources [16, 22].

Previous research used surface electromyography (sEMG) to evaluate postural control under DT condition [24–27]. A cognitive task seems to reduce muscle activity quantified as normalized signal amplitude during an ankle movement task [26] and a balance recovery one [25]. However, co-contraction index seems not to vary between conditions in adult healthy participants [27]. Fatigue can reverse previous relationship, in fact after a workload, muscle activity under DT increases [24]. On the contrary, difficult of the cognitive task did not influence sEMG parameters [26]. In OA reduction in sEMG magnitude is more pronounced than in their younger counterpart [25] in both agonist and antagonist muscles [25], suggesting that less attentional resources for balance are available.

The effect of different types of cognitive tasks on muscle excitation and DT cost remains understudied. Moreover, heterogeneity between postural balance tasks and their difficulties can hide the true effect. Thus, the aim of this study is to evaluate the effect of different types of cognitive tasks on muscle excitation and static postural control in young and older adults in an upright posture maintenance task. Our hypothesis is that an interaction effect between age and type of task would be present for stabilometric and muscle excitation variables.

## Material and methods

### Participants

The Sport and Exercise Medicine Division, University of Padova provided two databases for sample recruitment of OA and YA. Records were selected according to a database specific computer-generated list of random numbers and then potential participants were contacted by phone and they were asked to participate in an anamnesis session. Exclusion criteria were: (i) uncorrected visual impairments, (ii) lower limbs orthopaedic surgery in the 6 months prior to the study, (iii) use of medication that could influence postural control, (iv) neurological disorders, (v) cognitive impairments, vi) history of falls in the previous 6 months. Moreover, participants were eligible if they were able to stand upright for 10 minutes without supports (self-reported). At the end of this process, 31 YA (17 women, age 22 ± 4 yr, range 18–26) and 30 OA (19 women, age 72 ± 4, range 65–84) were enrolled to take part to the study. All participants completed an informed consent process before starting experimentation. Sample size was selected according with Bergamin et al. [13]. In the first session, one researcher administered a medical history questionnaire and the Mini-Mental State Examination (MMSE) [28]. Cut off value for MMSE was 27. In eligible participants, height and body mass were measured.

### Procedure

In the second session participants carried out the following tasks, for 30 s each: open (OE) and closed (CE) eyes, spatial-memory brooks’ test (SMBT) [29], counting backwards aloud test (CBAT) and mental arithmetic task (MAT) [21]. Participants were asked to stay as still as possible [30] while performing a modified Romberg Test [31]: upright stance, gaze at the target on the wall, arms along the body, joined heels and tips of the feet spread apart of thirty degrees. Before starting, the target was set at eye level. The order of experimental conditions was randomized to prevent learning effect. Five minutes rest between conditions was planned to reduce potential fatigue effect. Three trials for each condition were performed and the mean value for each variable was considered.

### Experimental dual tasks

#### Spatial-memory Brooks’ test

Before starting, an empty 4 by 4 matrix with only the number one in position 2,2 was shown. A computerized audio track, randomly selected from 12 available, indicated the spatial placing for number from 1 to 8. Numbers can be placed to the right, left, up or down respect to the previously filled square, but they cannot overlap themselves or be place outside the matrix. At the end of the trial participants had to fill the matrix with the numerical sequence. Two familiarization attempts were planned before procedure began. Individual score was determined counting correct answers, maximal score was 7.

#### Counting backwards aloud test

Participant was asked to count backward aloud by one, as fast and as accurately as possible, with a clear pronunciation from a randomly selected number from 90 to 100. Individual score was determined by counting mistakes [32].

#### Mental arithmetic task

A computerized audio-track enunciated nine arithmetic operations mixed between additions and subtractions with a pause of 2 s between them. A software randomly selected the digit sequence so that partial and final algebraic sums were always positive natural numbers. Participants were asked to mentally and wordlessly solve numerical series and to return the result of the trial. Score was a binary variable, “correct” or “wrong”.

### Outcomes assessments

#### Electromyography recording and processing

After appropriate cleaning and preparation of the skin sEMG probes (FREEEMG, BTS Bioengineering, Padova) were applied bilaterally, according with Blanc’s guidelines [33], over Tibialis anterior (TA), Lateral Gastrocnemius (GL), Peroneus Longus (PL), Erector Spinae Longissimus (ES). The 8-channel surface electromyographic system was used with a frequency of acquisition of 1000 Hz for each channel. Signals were band pass filtered between 10 and 450 Hz with a 5th order Butterworth filter and full wave rectified. The root mean square (RMS) of the signal over the entire trial (on a 50 ms window) was computed by low-pass filtering the signals with a 4th order Butterworth filter and a cut off frequency of 5 Hz [34]. The mean RMS between left and right leg muscles and left and right ES muscles were used. To allow comparisons results are expressed as percentage of the muscle activity during OE condition [35]. Data were processed using Matlab **(**MATLAB and Statistics Toolbox Release 2012b, The MathWorks, Inc., Natick, Massachusetts, United States).

#### Stabilometric variables recording and processing

Ground reaction forces were recorded by a force platform (FP4060-10, Bertec Corporation, Columbus, OH) with a sampling frequency of 960 Hz, fixed to the floor 3 m away from walls and 5 m from the target. Foot placement was standardized as recommended by previous findings [31, 36]. A set of parameters was chosen according with the literature [36–38]. The following variables were taken for the analysis: center of pressure (CoP) excursion (path), CoP velocity (CoPv), sway area (SA)[31], root mean square of CoP displacement (RMS) and 50% and 95% of power frequency [31]. CoPv was defined as the mean velocity of CoP during the entire trial. The SA estimate the area enclosed by the CoP path per unit of time, calculated summing up the area of the triangles formed by two consecutive points on the CoP path and the mean CoP [31]. The frequency, below which 50% and 95% of the total power frequency was found, are indicated as 50% and 95% frequency. The RMS represent the standard deviation respect to the mean displacement of the CoP [31]. Velocity, displacement and frequencies measures components along anterior–posterior (z) and medio-lateral (x) axis are reported. Data were processed using Matlab **(**MATLAB and Statistics Toolbox Release 2012b, The MathWorks, Inc., Natick, Massachusetts, United States). The force plate was synchronized with the dual task audio track to allow for an exact 30 s duration of the task and recording.

### Statistical analysis

Sociodemographic characteristics were compared through T-test. Chi-square was used to compare results of the cognitive task (treated as dichotomous) and group composition.

Due to highly skewed distributions, the based-10 logarithm (Log) was calculated for each variable. To assess the need for correction for anthropometric characteristics, stabilometric parameters were plotted against height and body mass. Visual analysis and a significant Pearson product moment correlation with *r* > 0.7 were used as criteria for normalization. No one of the parameters met the requirement for correction.

The percentage differences between the Log values of each condition (CE, CBAT, MAT, SMBT) and control one (OE) were computed and used for the statistical analysis. Normality of the data was assessed via Q-Q plot and Shapiro–Wilk. Mild violations of this assumption were allowed due to the robustness of the model. Two-way split-plot ANOVA was used with group membership as between subject factor (2 levels) and type of task as within factor (5 levels). Box test was used to check equality of co-variance matrix with an α = 0.01. For all ANOVAs, sphericity was tested with the Mauchly test for the dependent variables within group analysis. If the assumption of sphericity was violated, the correction of Greenhouse–Geisser was applied. Levene’s test was used to check the assumption of equality of variances for between group analyses. The presence of outliers was evaluated via residuals. To follow-up significant interaction effect, repeated measure ANOVA was used to test for main effect for task within each group and independent *T* test was used to test main effect for group. Effect size for any main factor and interaction was calculated and presented by partial *η*^2^ (*η*^2^*p*).

We decided to apply Bonferroni’s correction only to pairwise comparisons to follow up a significant main effect for condition. Hence, we reported the corrected values. However, as suggested by one of the reviewers, we calculated the Bonferroni’s level of significance accounting for 139 tests (45 Fisher tests and 94 pairwise comparisons), that is 0.00037. We will discuss the finding accounting this value for the calculation, though we are not using it as a binary criterion to determine significance. For this reasons, exact *p* value are reported.

Statistical analysis was performed with R [R Core Team (2018)]. Results are expressed as mean (SD).

## Results

In the present section, we are using the abbreviation of the experimental condition (CE, CBAT, MAT, SMBT) to indicate the percentage differences of the Log raw values respect to OE condition.

### Sociodemographic characteristics and cognitive performance

The OA group was composed by 30 participants (19 women, 63%) and the YA group by 31 (17 women, 55%). The height was significantly higher in YA than OA. Moreover, OA has a significantly higher BMI than YA. Groups did not differ on cognitive performance. Sociodemographic characteristics and cognitive performance error counts are exposed in Table 1.

**Table 1:** Demographic characteristics of the study participants and DT results

Variables	OA	YA	*p*
Demographic characteristics			
Participants (% female)	30(63%)	31(55%)	/
Age (years)	72.70(5.38)	22.42(2.45)	/
Weight (Kg)	67.73(14.28)	67.85(13.73)	0.9731
Height (cm)	1.63(0.10)	1.71(0.10)	0.001*
BMI (Kg m^−2^)	25.25(3.75)	22.96(3.57)	0.014*

DT errors	OA	YA	*p*
CBAT (number)	6	2	0.117
MAT (number)	7	4	0.289
SMBT (number)	12	7	0.142

Results are expressed as mean (sd). An asterisk (*) is used to highlight *p* < 0.05. Age was rounded down to the nearest whole value

*n* number of cases, *sd* standard deviation, *p p* value, *OA* Older Adults, *YA* Young Adults, *W* Women, *M* Men, *DT* Dual task, *CBAT* counting backward aloud test, *MAT* mental arithmetic task, *SMBT* spatial memory Brooks’ test 4

### Stabilometric analysis

**Fig. 1:**
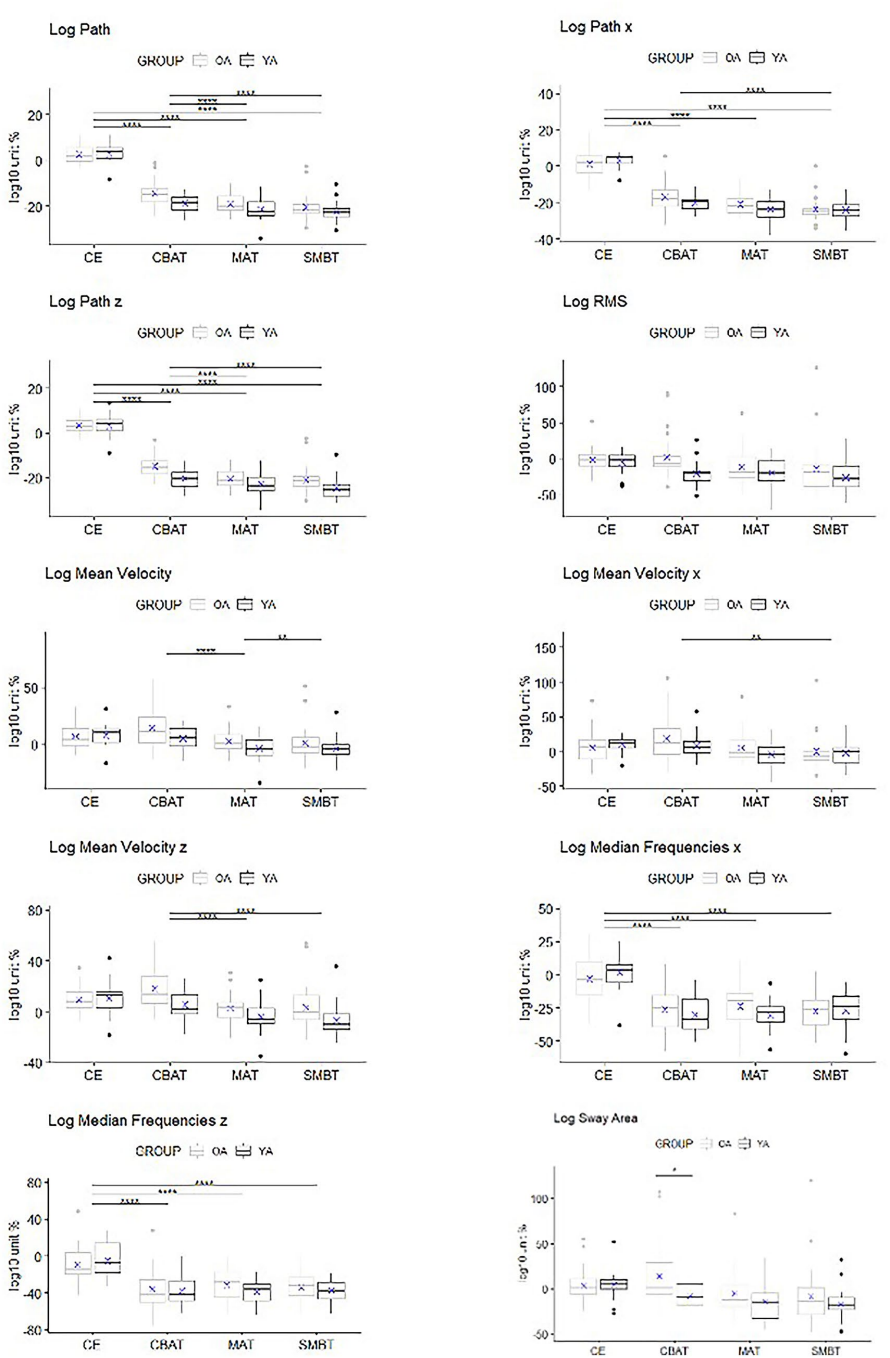
Boxplots with superimposed the mean (*X*) for stabilometric variables. In the sway area graph, only the significance within CBAT condition is reported to not add confusion to the figure. For the complete results of this variable, please refer to the homonymous section. *, *p *< 0.05, **, *p *< 0.01, ***, *p *< 0.001, ****, *p *< 0.0001**. ***OA* Older Adults, *YA*: Young Adults, *CE* closed eyes, *CBAT* counting backward aloud test, *MAT* mental arithmetic task, *SMBT* spatial memory Brooks test

The results of split-plot ANOVA on posturographic variables are extensively reported in Table 2. There was a statistically significant interaction between tasks and groups only for Log Sway Area (*F*_(2.55, 106.92)_ = 4.03, *p *= 0.01 *η*^2^*p *= 0.09). Following up main effects, it emerged that OA (mean =  − 8.79, sd = 16.69) differed significantly from YA (mean = 13.86, sd = 33.66) only in the CBAT condition (*t*_(46)_ = 3.491, *p *= 0.01, *d* = 0.209). Moreover, a significant effect for task was present for both, the OA (*F*_(2.33, 55.85)_ = 10.71, *p *< 0.0001, *η*^2^*p *= 0.31) and the YA (*F*_(3, 54)_ = 7.03, *p *= 0.0005, *η*^2^*p *= 0.28). For the OA, there was statistically significant differences between: CBAT vs MAT (*p *= 0.011), CBAT vs SMBT (*p *= 0.0002). For the YA, there was statistically significant differences between: CE vs MAT (*p *= 0.003), CE vs SMBT (*p *= 0.002). A main effect for task emerged for all variables; Table 3 reports pairwise comparisons (see Fig. 1). Only Log 95% frequencies z showed a main effect for group (*F*_(1,53)_ = 4.47, *p *= 0.04 *η*^2^*p *= 0.08). In all the DT condition, OA had a greater negative difference (respectively, *p *= 0.002, *p *= 0.04, *p *= 0.02) from control condition if compared to YA.

**Table 2:** Analysis of stabilometric variables

Stabilometric variables	Groups	CE	CBAT	MAT	SMBT	dof, dofE, *F*	Task × group	Partial eta squared	dof, dofE, *F*	Task	Partial eta squared	dof, dofE, *F*	Group	Partial eta squared
Log path	OA	2.62 (3.79)	− 14.34 (5.71)	− 19.06 (4.21)	− 20.45 (5.92)	3, 162, 1.12	0.34	0.02	3, 162, 521.85	*p *< 0.0001*	0.91	1, 54, 1.16	0.286	0.02
	YA	2.46 (4.36)	16.46 (5.24)	− 20.17 (4.91)	− 21.10 (4.91)									
Log path *x*	OA	1.22 (6.63)	− 16.88 (8.38)	− 20.84 (5.90)	− 23.63 (6.75)	3, 162, 2.13	0.10	0.04	3, 162, 393.17	*p *< 0.0001*	0.88	1, 54, 0.22	0.64	< 0.01
	YA	2.68 (4.11)	− 18.89 (4.91)	− 22.46 (5.89)	− 22.79 (5.50)									
Log path *z*	OA	3.67 (3.45)	− 14.60 (4.97)	− 20.20 (4.18)	− 20.93 (6.43)	3, 162, 0.91	0.44	0.02	3, 162, 462.84	*p *< 0.0001*	0.90	1, 54, 2.40	0.13	0.04
	YA	2.80 (5.26)	− 17.19 (6.35)	− 21.19 (5.23)	− 22.88 (5.94)									
Log sway area	OA	3.75 (18.66)	13.86 (33.66)	− 5.29 (25.28)	− 8.75(34.58)	2.55, 106.92, 4.03	0.01*	0.09	2.55, 106.92, 13.02	*p *< 0.0001*	0.24	1, 42, 2.83	0.10	0.06
	YA	4.43 (16.96)	− 8.79 (16.69)	− 12.72 (22.88)	− 16.59(18.94)									
Log RMS	OA	− 1.77 (15.95)	2.51 (30.49)	− 11.69 (24.11)	− 13.40(37.76)	2.36, 110.85, 0.64	0.58	0.01	2.36, 110.85, 4.99	0.006	0.10	1, 47, 0.02	0.902	< 0.01
	YA	8.21 (58.78)	− 1.02 (66.29)	− 9.09 (29.70)	− 16.76(28.95)									
Log mean velocity	OA	5.23 (12.68)	15.57 (18.29)	3.06 (11.65)	1.14(15.90)	2.5, 134.9, 1.42	0.24	0.03	2.5, 134.9, 22.93	*p *< 0.0001*	0.30	1, 54, 0.73	0.40	0.01
	YA	6.84 (10.47)	11.06 (14.23)	− 0.13 (12.23)	− 0.88(12.93)									
Log mean velocity *x*	OA	5.64 (21.77)	20.86 (34.28)	6.55 (21.95)	− 0.04(25.91)	2.6, 140.49, 1.85	0.15	0.03	2.6, 140.49, 15.34	*p *< 0.0001*	0.22	1, 54, 0.36	0.55	< 0.01
	YA	9.13 (12.73)	14.60 (22.16)	− 0.26 (17.23)	1.69(20.23)									
Log mean velocity *z*	OA	9.10 (11.07)	19.73 (18.68)	3.63 (12.87)	3.53(18.61)	3, 162, 0.95	0.42	0.02	3, 162, 24.80	*p *< 0.0001*	0.32	1, 54, 1.55	0.22	0.03
	YA	8.99 (13.83)	14.21 (18.32)	0.80 (14.84)	− 2.10(16.60)									
Log 95% frequencies *x*	OA	− 23.62 (47.19)	− 52.12 (33.70)	− 43.11 (34.22)	− 52.79(46.39)	1.81, 97.71, 1.30	0.28	0.02	1.81, 97.71, 0.12	0.87	< 0.01	1, 54, 2.93	0.09	0.05
	YA	− 33.63 (187.33)	7.60 (116.93)	− 15.37 (108.48)	− 13.50(120.44)									
Log 95% frequencies *z*	OA	− 14.37 (49.18)	− 82.67 (52.12)	− 74.15 (60.17)	− 74.17(70.11)	1.72, 91.03, 3.47	0.05	0.06	1.72, 91.03, 1.73	0.19	0.03	1, 53, 4.47	0.04*	0.08
	YA	− 36.94 (161.10)	− 28.87 (73.19)	− 33.36 (84.95)	− 25.40(76.57)									
Log median frequencies *x*	OA	− 14.13 (60.78)	− 33.89 (46.70)	− 34.01 (55.43)	− 37.39 (47.79)	1.9, 100.79, 0.26	0.758	< 0.01	1.9, 100.79, 13.10	*p *< 0.0001*	0.20	1, 53, 2.28	0.14	0.04
	YA	1.55 (46.45)	− 17.66 (24.39)	− 23.96 (25.48)	− 19.55 (20.63)									
Log median frequencies *z*	OA	− 18.23 (51.49)	− 42.69 (40.17)	− 38.26 (40.79)	− 42.35 (45.49)	1.75, 94.46, 0.83	0.43	0.02	1.75, 94.46, 14.82	*p *< 0.0001*	0.22	1, 54, 3.04	0.09	0.05
	YA	6.54 (65.47)	− 30.48 (27.83)	− 24.39 (28.63)	− 29.71 (30.29)									

Emg variables	Groups	CE	CBAT	MAT	SMBT	dof, dofE, *F*	Task × group	Partial eta squared	dof, dofE, *F*	Task	Partial eta squared	dof, dofE, *F*	Group	Partial eta squared
Log TA	OA	13.76 (29.04)	5.43 (29.53)	− 6.84 (19.46)	− 3.91 (21.14)	2.04, 116.45, 1.45	0.24	0.02	2.04, 116.45, 8.26	*p *< 0.0001*	0.13	1, 57, 0.02	0.89	< 0.01
	YA	5.01 (32.37)	5.37 (25.28)	0.20 (28.50)	− 4.35 (17.20)									
Log GL	OA	2.50 (10.07)	4.85 (13.68)	3.76 (11.18)	1.65 (13.09)	3, 171, 2.36	0.07	0.04	3, 171, 1.29	0.28	0.02	1, 57, 1.29	0.26	0.02
	YA	3.49 (18.28)	− 2.37 (13.91)	− 0.96 (13.28)	− 1.04 (12.03)									
Log PL	OA	0.17 (17.03)	− 1.26 (19.83)	− 3.23 (16.21)	− 3.95 (17.59)	3, 171, 0.37	0.77	< 0.01	3, 171, 1.91	0.13	0.03	1, 57, 1.02	0.32	0.02
	YA	− 4.20 (19.81)	− 2.40 (13.80)	− 4.30 (14.72)	− 8.00 (20.49)									
Log ES	OA	0.34(7.34)	4.76 (12.36)	0.65 (12.91)	− 0.86 (12.21)	3,168, 0.21	0.89	< 0.01	3, 168, 6.29	*p *< 0.0001*	0.10	1, 56, 0.03	0.87	< 0.01
	YA	− 0.52(10.38)	3.18 (10.21)	− 0.52 (11.98)	− 0.54 (10.64)									

Results are expressed as mean (sd). An asterisk (*) is used to highlight *p* < 0.05 *p* are visualized under the columns Task × Group, Task, Group that represent the interaction and main effects respectively

*n* number of cases, *sd* standard deviation, *dof* degrees of freedom, *dofE* degrees of freedom for the error term, *Log* Logarithm, *OA* Older Adults, *YA* Young Adults, *W* Women, *M* Men, *CBAT* counting backward aloud test, *MAT* mental arithmetic task, *SMBT* spatial memory Brooks’ test, *TA* Tibialis Anterior, *GL* Gastrocnemius Lateralis, *PL* Peroneus Longus, *ES* Erector Spinae

**Table 3:** Pairwise comparisons for task

Variables	CE vs CBAT	CE vs MAT	CE vs SMBT	CBAT vs MAT	CBAT vs SMBT	MAT vs SMBT
Log path	< 0.0001*	< 0.0001*	< 0.0001*	< 0.0001*	< 0.0001*	1
Log path *x*	< 0.0001*	< 0.0001*	< 0.0001*	0.10	< 0.0001*	1
Log Path *z*	< 0.0001*	< 0.0001*	< 0.0001*	< 0.0001*	< 0.0001*	1
Log RMS	1	0.12	0.54	0.092	0.023*	1
Log mean velocity	0.05	1	1	< 0.0001*	< 0.0001*	1
Log mean velocity *x*	0.02	1	0.6	0.04*	0.002*	0.41
Log mean velocity *z*	0.042*	0.44	0.92	< 0.0001*	< 0.0001*	1
Log median frequencies *x*	< 0.0001*	< 0.0001*	< 0.0001*	1	1	1
Log median frequencies *z*	< 0.0001*	< 0.0001*	< 0.0001*	1	1	1
TA	0.68	0.004*	0.01*	1	0.06	0.97
ES	0.18	1	1	0.05	0.03*	1

*p* value for pairwise comparison to follow up main effect for task

*OE* open eyes, *CE* closed eyes, *CBAT* counting backward aloud test, *MAT* mental arithmetic task, *SMBT* spatial memory Brooks test, *TA* Tibialis Anterior, *ES* Erector Spinae

### EMG analysis

**Fig. 2:**
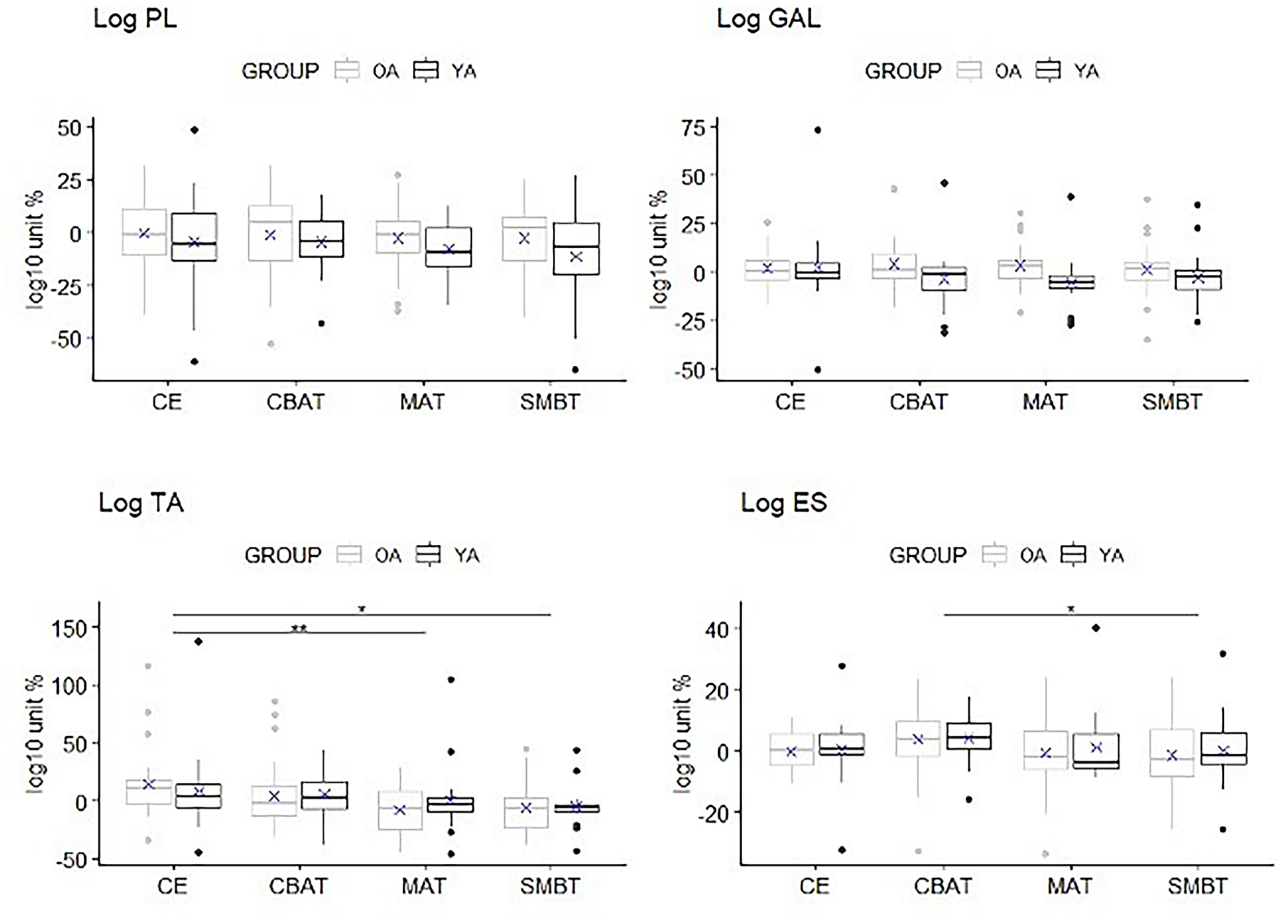
Boxplots with superimposed the mean (*X*) for electromyographical variables. *, *p *< 0.05, **, *p *< 0.01, ***, *p *< 0.001, ****, *p *< 0.0001**. ***OA* Older Adults, *YA* Young Adults, *CE* closed eyes, *CBAT* counting backward aloud test, *MAT* mental arithmetic task, *SMBT* spatial memory Brooks test. *TA* Tibialis Anterior, *GL* Gastrocnemius Lateralis, *PL* Peroneus Longus, *ES* Erector Spinae

Statistical interaction between age and condition was not found in the considered muscle, as main effect for age group. However, TA and ES showed a main effect for condition, respectively *F*_(2.04,116.45)_ = 8.26, *p* = 0.0004 *η*^2^*p* < 0.01, and *F*_(3,168)_ = 6.29, *p* = 0.0005, *η*^2^*p* = 0.01. Table 3 reports pairwise comparisons (see Fig. 2).

## Discussion

### Main findings

This study aimed to evaluate the effect of different types of cognitive tasks on postural control and muscle excitation of the lower limb and the trunk, between OA and YA. It was hypothesized that interaction effect should be present between age and type of secondary task in all the variables, however the main finding is that interaction occurred only for Log SA. The portion of variance explained by interaction, excluding the main effect, is 9% (*η*^2^*p* = 0.09). Moreover, the *p* value for interaction does not exceed the Bonferroni’s level of significance (*p* = 0.01 vs *p* = 0.00037). Type of task seems to act in an independent manner, disregarding the age of participants. These results were in agreement with previous findings: OA were able to manage the complexity of DT as the younger people in a stationary condition [13, 14], despite a dynamic secondary task would have shown a better discriminant capacity, being more challenging for the postural control system [39].

However, considering that, Bonferroni adjustment tends to overcorrect the level of significance and that multiple authors are against its use [40], our intent is to explain why the interaction was occurring. The presence of this effect was justified by the fact that OA increased their SA respect to control condition during CBAT and decreased it during MAT and SMBT. On the contrary, SA in young adults increased during CE, and differed from MAT and SMBT. One possible reason could be that verbalization increased respiratory acts, influencing CoP displacement [41]. In support to this hypothesis, we reported significantly higher ES muscle excitation in CBAT respect SMBT (the difference between CBAT and MAT approximate significance), similarly to the augmented ES muscle excitation found by Hodges et al. [41] during increased tidal volume condition. Generally, OA tends to respond to perturbation increasing co-contraction [5]. Whereas DT interferes with this mechanism, postural sway will be over-increased and interaction effect detected in SA could explain this response. Co-contraction is a function of agonist–antagonist muscle excitation [42]. As GL activity did not vary according to the task (*p* = 0.28) while TA did (*p* =  < 0.0001), it could be speculated that co-contraction in the lower leg was reduced. In considering findings of muscle activity, it is necessary to consider that even if the part of variance explained is low, they are quite consistent as they reach significance even after the correction for family-wise error rate. In agreement with our results, others reported a reduction of TA excitation during DT [26], but without difference when increasing the task complexity. However, it has to be considered that types of tasks were extremely different, as participants in the reference study performed an ankle movement while sitting.

The authors of this paper were not able to explain why the interaction was occurring only for SA. Normally postural sway parameter accounts for a certain degree of redundancy [43, 44]. Evaluating the mean of the parameter with a dimensional equation similar to SA, as mean velocity (and its components), you will see a similar behaviour. Given that, squaring the length component “increased” his importance in the computation. A second interpretation could be that that interaction is due to chance and parameter only present a main effect for task (as stated above in the first paragraph).

A significant main effect for task was always present in the stabilometric parameters except for Log 95% frequencies and Log RMS, with η^2^p ranging from 0.20 to 0.91. Considering that RMS is linked to total power of the spectrum, [43] it can explain the similar trend as 95% frequency. Time-domain parameters reflected partially the previous results of Bergamin et al. [13]. All parameters related to CoP path and RMS were significantly higher in ST than DT, even if the effect of task was more accentuated on path length and its components. A possible explication is that performing an external task forced it to be automatic (participants are no more concentrated to stand “as still as possible” trying to improve the static postural control performance [45]). According to previous literature, the instruction “stand as still as possible” improve reliability of the measure and the performance during the single task of postural control [30]. This phenomenon probably occurs because it reduces the degree of accommodation during “stand quietly” or no instruction trials. The DT conditions permits to obtain automaticity and no accommodation at the same time, improving the performance. From another perspective, performing a secondary task can produce undesired movements due to cognitive load, e.g., oculomotor activity [46], and the postural control system can organize itself to prevent these type of movements increasing co-contraction or acting to increase stability. Moreover, performing a secondary task has been demonstrated in reducing the exploratory movement of the feet [47].

Literature about frequencies showed that they could be useful to discern between visual, vestibular and somatosensory system actions [48]. In the present experiment, 95% percent frequencies did not present a main effect for task or condition. Considering a potential role in discerning between postural control systems, the motivation can lie in the facilitating effect of DT. If participant balance condition were not threatened and if one system did not overcome the others, the parameter should not change. Moreover, considering this interpretation, our results showed agreement with those of Prieto et al. [49], meaning that at least the same set of parameters should be used under DT conditions to better capture CoP characteristics.

In contrast with previous findings [49, 50], a main effect for group was found only for Log 95%. However, considering the p value and small effect size (*η*^2^*p* = 0.08) we can assume that is negligible. This finding is not surprising as we computed the differences from the control condition, while the reported studies analyse the raw value. It is well documented in the literature that static postural control worsens with age, however OA and YA respond in a similar manner to the different task, if we account to the baseline.

About muscle activation, our results seemed to be in contrast with current literature which reported an increased activation in OA than YA [25]. However, that investigation analysed a balance recovery task while we analysed a balance maintenance task, and a direct comparison could generate misleading interpretations. Additionally, GL did not show any effect. This muscle is the main responsible for anterior–posterior oscillations [51] and according to prioritization and hazard estimation theory [12], postural control system could have maintained constant the muscle activity preventing the risk of fall.

In summary, DT affected participant’s postural control and TA and ES muscle excitation, inducing a facilitating effect. An interaction effect was founded on SA in OA, tending to increase for CBAT condition but lowering during other DT respect to ST. Conversely, in YA, SA decreased in all DT conditions. Normalization to control condition eliminates all the other differences due to group membership. Muscle excitation did not show a homogeneous behaviour; in fact, GL and PL remained unaltered, while muscle activity changed for TA and ES.

### Limitations

Stochastic parameters should be included to describe CoP displacement [52], but the longer testing time needed [38] could prevent the administration of five experimental conditions, potentially generating other bias (e.g., muscle fatigue in the elderly). Another potential criticism was that EMG normalization on OE condition was not equivalent to normalizing on the reference task for each muscle; however, during OE condition muscles work in near isometric manner and, in this way, results could be expressed as fraction of OE. Moreover, the normalization for OE condition was specific for the postural task performed during DT condition. With respect to EMG analysis, it should be mentioned that the detection of the timing of skeletal muscles activation prior to the extraction of any parameters from the EMG signal plays a significant role. Due to high signal-to-noise ratio required for accuracy in estimation, the detection of muscle activity becomes even more difficult [53] for myoelectric weak and noisy signals, such as those recorded during prolonged low-level sustained contractions (i.e., postural activity). In the present contribution a single threshold method was applied, which is more susceptible to the background noise and the environmental interferences. Asking to participant to stand as still as possible could be a limitation itself, creating a dual task condition in the reference task. However, using the “stand quietly” instruction may result in an accommodating posture due to lack of instructions.

## Conclusion

The present study indicated that DT improves static postural control, probably increasing automaticity of the motor task. The most challenging experimental condition is CBAT, due to verbalization. Accounting for the baseline level, the performance of YA and OA varies in the same manner within the different condition for all the variables except sway area. Moreover, during a postural maintenance task muscle excitation is not different between OA and YA. Shank muscles showed a different response to experimental condition with only TA influenced by the DT, but not by task complexity. We can conclude that YA and OA tend to respond in the same manner to DT, exhibiting a facilitating effect in respect to single task condition.
